# Engineering Human Circulating Monocytes/Macrophages by Systemic Deliverable Gene Editing

**DOI:** 10.3389/fimmu.2022.754557

**Published:** 2022-05-18

**Authors:** So Yoon Lee, Javier Fierro, Jake Dipasquale, Anthony Bastian, An M. Tran, Deawoo Hong, Brandon Chin, Paul J. Nguyen-Lee, Sarah Mazal, Jamil Espinal, Tima Thomas, Huanyu Dou

**Affiliations:** ^1^ Department of Molecular and Translational Medicine of Paul L. Foster School of Medicine, Texas Tech University Health Sciences Center at El Paso, El Paso, TX, United States; ^2^ Biomedical Sciences Graduate School, Texas Tech University Health Sciences Center at El Paso, El Paso, TX, United States

**Keywords:** human circulating monocytes-derived macrophages, systemic deliverable gene-editing, IL-4, plasmid DNA, immune regulation

## Abstract

Delivery of plasmid DNA to transfect human primary macrophages is extremely difficult, especially for genetic engineering. Engineering macrophages is imperative for the treatment of many diseases including infectious diseases, cancer, neurological diseases, and aging. Unfortunately, plasmid does not cross the nuclear membranes of terminally differentiated macrophages to integrate the plasmid DNA (pDNA) into their genome. To address this issue, we have developed a core-shell nanoparticle (NP) using our newly created cationic lipid to deliver the anti-inflammatory cytokine IL-4 pDNA (IL-4pDNA-NPs). Human blood monocyte-derived macrophages (MDM) were effectively transfected with IL-4pDNA-NPs. IL-4pDNA-NPs were internalized in MDM within 30 minutes and delivered into the nucleus within 2 hours. Exogenous IL-4 expression was detected within 1 - 2 days and continued up to 30 days. Functional IL-4 expression led to M2 macrophage polarization *in vitro* and in an *in vivo* mouse model of inflammation. These data suggest that these NPs can protect pDNA from degradation by nucleases once inside the cell, and can transport pDNA into the nucleus to enhance gene delivery in macrophages *in vitro* and *in vivo*. In this research, we developed a new method to deliver plasmids into the nucleus of monocytes and macrophages for gene-editing. Introducing IL-4 pDNA into macrophages provides a new gene therapy solution for the treatment of various diseases.

## Introduction

Plasmid gene editing provides a novel therapeutic strategy to cure many diseases including infectious diseases, cancer, neurological diseases, and aging (1–8). This approach prevents immune toxicity and improves the efficacy and safety of immune manipulation. Systemic deliverable nano-based genetic immune regulation has been widely studied (9–11). However, macrophages are non-dividing cells that are extremely difficult to transfect for plasmid gene editing. As professional phagocytes, monocytes/macrophages can effectively recognize, neutralize, and degrade foreign molecules, making it significantly challenging to apply gene therapy that requires the introduction of “foreign molecules” into the cell. Currently, most gene delivery techniques fail to deliver plasmid to macrophages because of the ability to clear “foreign molecules”, as well as cellular membrane barriers that limit entry into non-dividing nucleus. Developing an effective delivery system to overcome phagocytosis and nuclear membrane barriers in macrophages is critical for macrophage-targeted gene therapy.

Monocytes/macrophages are present in almost all tissues of the human body and play a central role in immune responses. They can be rapidly polarized to undergo morphological, functional, and biochemical changes in response to local and systemic environmental stimuli (12–15). These cellular mechanisms and signaling pathways regulate immune responses to control recovery, repair and regeneration (16). Macrophages, in particular, participate in both innate and adaptive immune responses to promote angiogenesis, inflammation remodeling, and support recovery. During pathological processes, macrophages exhibit two main phenotypes, classically activated (proinflammatory M1) and alternatively activated (anti-inflammatory and tissue-remodeling M2) (17, 18). In the presence of inflammatory stimuli, M1 macrophages upregulate the expression of a number of proinflammatory genes such as IL-1β, IL-6, IL-12 and TNF-α (19–21). M2 macrophages, on the other hand, contribute to tissue repair, remodeling, and oncogenesis. Anti-inflammatory cytokines such as IL-10, IL-4 and IL-13 repolarize macrophages from M1 to M2. M1/M2 macrophage polarization is reversible, therefore, targeting macrophages to treat disease is a promising approach to developing new immunotherapies (22, 23). The M1-M2 paradigm is commonly associated with properties of mature macrophages, but activation takes place in the extended macrophage family, including monocytes, myeloid-derived dendritic cells and multinucleated giant cells. In tissues, all these events combine to produce a resultant phenotype, and, though useful for the sake of understanding, any sort of hierarchy or order does not represent the biology of the cells. Cytokines and antibodies can be used to regulate macrophages. However, short half-lives causes multiple dosing regimens that can trigger immune overreaction and toxicity. This prevents clinical uses of macrophage-targeted gene therapy and vaccination. Genetic manipulation of macrophages by introducing exogenous genes has great potential to treat various diseases (24, 25) by regulating immune responses, remodeling injured tissue, and altering the tumor environment.

Nano-based plasmid delivery has become an attractive technique due to its numerous advantages including packaging of large molecular weight Adeno-associated Virus (AAV) plasmid targeting of specific tissue/cell types, ability to cross both cytoplasmic and nuclear membranes, transfection of non-dividing cells, and diversifying functionalization. Nevertheless, the significant challenge is to deliver the gene across both cytoplasmic and nuclear membranes for nuclear ingress in non-dividing cells. Moreover, the free genes rarely reach the cells and or diseased tissues due to degradation, inefficient cellular internalization, and lack of targeting properties (26). In fact, non-viral delivery techniques including nanoformulations and cell-penetrating peptides, have shown significant benefits (27, 28). Our previous work synthesized a cationic lipid using branched low molecular weight polyethyleneimine (PEI) and glycidyl hexadecyl ether (GHE) to create nanoformulations to deliver IL-4 plasmid (29) in cell lines. In this study, we further developed NPs formed with this cationic lipid shell and a Poly(D,L-lactide-co-glycolide) (PLGA) core. This core-shell NP encapsulated IL-4/GFP pDNA empowered effective transfection in MDM and in a mouse model. As a proof-of concept, full length IL-4 plasmid DNA (IL-4pDNA) loaded in NPs were efficiently internalized by MDM within 30 minutes and transported into the nucleus within 2 hours, resulting in significant exogenous IL-4 expression in MDM. In addition, IL-4pDNA-NPs repolarized LPS-induced inflammatory macrophages from M1 into M2 *in vitro* and *in vivo*. These findings suggest that this new delivery system was capable of transporting plasmids into the nucleus of cells for regulating macrophage functions, providing a new gene therapeutic platform for the treatment of various diseases.

## Methods

### PEI Lipid/PLGA NPs Formulation

GHE (MW = 298 g/mol; Sigma-Aldrich, St Louis, MO) and low molecular weight branched PEI (MW = 800 g/mol; Sigma-Aldrich, St Louis, MO) were mixed in a glass vial at a molar ratio of 8:1. The vial was sealed and the samples were stirred at 300 rpm for 24 hours at 40°C. Afterward, the sample was cooled at room temperature and the obtained white solid was broken down into a fine powder and stored at 4°C.

NPs were prepared by a modified solvent extraction/evaporation method. Briefly, 25 mg of PEI lipid, 15 mg of Poly(D,L-lactide-co-glycolide) (50/50) (PLGA; LACTEL Absorbable Polymers, Birmingham, AL) and 2 mg of 1,2-Dimyristoyl-sn-glycerol, Methoxypolyethylene Glycol (DMG-PEG; NOF America, White Plains, NY) were dissolved in 0.5 mL of dichloromethane (Sigma-Aldrich, St Louis, MO). 0.3 mg Rhodamine 6G (Sigma-Aldrich, St Louis, MO) was added for intracellular trafficking experiments. The solution was mixed with 2.5 mL DD water then sonicated to obtain a fine emulsion. The emulsion was further stirred overnight at room temperature then filtered with an Amicon Ultra-4 centrifugal filter (molecular weight cutoff of 100 kDa; Sigma-Aldrich, St Louis, MO). The suspension was adjusted to 2.5 mL with DD water and stored at 4°C.

### Production of Plasmid to Make Human Il-4 Plasmid

The “Helper-free” AAV system pAAV-IRES-hrGFP was purchased from Agilent (Santa Clara, CA). Human IL-4 was cloned into pAAV-IRES-hrGFP to produce IL-4 plasmid DNA. The correct orientation of the inserted sequences and the resulting plasmid DNA were respectively checked by DNA sequencing analysis and agarose gel electrophoresis.

### Scanning Electron Microscopy (SEM)

Changes in the morphologies of the obtained NPs were observed by using a SEM (JEOL JSM-6010LA, Tokyo, Japan). In order to avoid break down of the liposomes and NPs, 50 µl of liposomes and NPs were placed onto the cover glass, and one drop of 2% of uranyl acetate (Cat. No. 22400, Electron Microscopy Sciences Hattfield, PA) was added for 15 minutes. The slide was then washed out with DI water, and the sample was kept in a vacuum desiccator to dry out for 24 hours. The dried samples were coated with gold/palladium using Gold/Palladium Target 57 mm diameter×0.1 mm thick (Electron Microscopy Sciences, Hatfield, PA) with research grade argon gas to observe SEM, which was operated at 15 kV with top view.

### Atomic Force Microscopy (AFM)

AFM was used to observe NPs morphology. NPs of 1.2 μg/ml in phosphate-buffered saline (PBS) were deposited onto glass slides and then mounted on the scanner of a stand-alone AFM microscope (Park Systems, Model: XE-100, Santa Clara, CA). Typical scan sizes were 1 - 10 μm at a resolution of 512 × 512 data points. Images were taken and then analyzed with Femtoscan software.

### Zetasizer

A zetasizer (Malvern Instruments, Model: Zetasizer Nano ZS, Malvern, United Kingdom) was used to measure the size distribution, zeta potential, and heterogeneity of the obtained NPs. For each sample, the NPs were tested three times at 25°C in double distilled water at a concentration 1 mg/mL.

### IL-4pDNA Encapsulation

NPs were loaded with human IL-4, human IL-4/GFP, mouse IL-4 or mouse IL-4/GFP pDNA (Sino Biological, Wayne, PA) based on the molar ratios of PEI nitrogen (N) to DNA phosphate (P) (N/P ratio). The following equation was used to determine the N/P ratio, where the molecular mass (MM) of the repeating unit for branched PEI is 43 Da, the number of primary nitrogen’s in the repeating unit of branched PEI is 4, and the MM of DNA used was 330 Da (30, 31).


$$\frac{N}{P}=\left(\frac{\;\mu g\;PEI}{\frac{MMof\;PEI\;Repeating\;Unit}{Primary\;Amines\;in\;PEI}\;}\right)/\left(\frac{\mu g\;DNA}{MM\;of\;DNA}\right)$$

DNA encapsulation by NPs was investigated by agarose gel electrophoresis. NPs were loaded with pDNA at different N/P ratios and ran on a 1% agarose gel for 30 minutes. pDNA was visualized with an Amersham Imager 680 blot and gel imager (GE Healthcare, Chicago, IL).

The IL-4/GFPpDNA-NPs were made at different N/P ratios in PBS. NPs suspension and IL-4pDNA solutions were prepared before each experiment at various N/P ratios. First, 1 μl of NP suspension (including of 0.1 μg of PEI) was mixed with 20 μl of PBS to disperse the NP suspension. Second, the prepared IL-4pDNA solution was added into the NP suspension with various amounts to meet the different N/P ratio. Third, IL-4pDNA solution was well mixed with the NP suspension, then the mixture was incubated for 30 minutes at room temperature before transfection.

### Macrophages Cultures and IL-4pDNA/GFP-NPs Transfection

For human monocyte-derived macrophage (MDM) cultures, peripheral blood CD14+ monocytes were cultured in DMEM supplemented with 10% heat-inactivated pooled human serum (Thermo Fisher Scientific, Waltham, MA), 1% penicillin-streptomycin (P/S), 2 mM L- Glutamine, and 1000 U/ml recombinant human MCSF at 37°C with 5% CO_2_. Monocytes were seeded into a standard tissue culture-treated plate at a density of 1 million cells per ml. Human peripheral blood monocytes were cultured up to 7 days, allowing differentiation into macrophages, then subsequently used for experiments.

For mouse bone marrow derived macrophage (BMM), the femur was collected from a Balb/C mouse (male 4-6 weeks) and the bone marrow was dissociated into single-cell suspensions. Bone marrow cells were cultured in DMEM with fetal bovine serum (10%), 1% penicillin-streptomycin (P/S), 2 mM L- Glutamine (Thermo Fisher Scientific, Waltham, MA), and 1000 U/ml highly purified recombinant macrophage colony stimulating factor (MCSF; R&D Systems, Minneapolis, MN) at 37°C with 5% CO_2_. Cells were allowed to differentiate into macrophages for 12-14 days, then samples were used for experimentation. All experiments were carried out in compliance with the ARRIVE guidelines. The animals used in this study were approved by Institutional Animal Care and Use Committee, Texas Tech University Health Sciences Center, El Paso.

For transfection of MDM, human IL-4pDNA/GFP-NPs were added to cells at a 1:1, 1:4, 1:10, 1:20 and 1:50 NPs/media ratio. The media was changed after four hours, and the cells were further cultured for 9 days. For transfection of BMM, 1.5 ng/mL of mouse IL-4pDNA/GFP-NPs were added to cells at a 1:20 NPs/media ratio. The media was changed after 4 hours, and the cells were further cultured for 5 days. Cells were imaged with a Nikon Ti fluorescence microscope and analyzed for GFP expression with Image Pro Plus software.

### Cell Viability Assay

MDM cells were seeded in a 96 well plate and transfected with IL-4pDNA/GFP-NPs for 24 hours. Non-treated cultures served as the control. After 24 hours, cell viability was assayed using the Quick Cell Proliferation Colorimetric Assay Kit (BioVision, Milpitas, CA) according to the manufacturer’s protocol. Samples were analyzed with the FlexStation 3 Multi-Mode Microplate Reader (Molecular Devices, San Jose, CA) at 450 nm, and the data collected was normalized to the controls. The experiment was repeated in triplicate.

### Co-Imaging With Scanning Electron Microscope (SEM) and Confocal Microscopy

To visualize intracellular transportation of IL-4pDNA-NPs, dual labeling of IL-4pDNA with YOYO-1 iodide (green, Thermo Fisher Scientific, Waltham, MA) at 1 dye molecule to 50 pDNA base pairs for 24 hours, and MDM with LysoTracker Red (Thermo Fisher Scientific, Waltham, MA) to label lysosome or CellBrite™ Fix555 Red (Biotium, Fremont, CA) to label cell membranes.

For better imaging of YOYO-IL-4pDNA-NPs uptake by macrophages to cross the membranes and enter the nucleus, the cultures were incubated with CellBrite™ Fix555 to label the cell membranes (red) and DAPI (blue) to illustrate the nucleus. The labeled cultures were treated to YOYO-IL-4pDNA-NPs (green) for live cell imaging by fluorescent microscopy automatically every 30 minutes. The live images at 10, 40, 100, 160, and 250 minutes were used to determine the interaction of YOYO-IL-4pDNA-NPs and cellular membranes.

To track intracellular transportation, MDM were cultured in an 8 well chamber slide and treated with YOYO-IL-4pDNA-NPs at a NPs/media ratio of 1:20. Then, YOYO-IL-4pDNA-NPs treated MDM were stained with LysoTracker Red and DAPI (blue) and analyzed at 0, 2, 2.5, 3, 3.5, and 4 hours post transfection. Cells were then fixed with 4% paraformaldehyde for co-imaging of SEM and confocal microscopy assay. *First*, the triple labeled 8 well chamber slides were scanned by a confocal microscope (Nikon Ti Eclipse microscope, Nikon Instruments Inc., Melville, NY). *Then*, to co-image with SEM, the fractured cells were obtained from confocal microscopy imaged slides by adhesion of cover glass. In brief, another cover glass was attached very tightly to the sample cover slips, then pulled out mechanically and dried in the vacuum desiccator for 24 hours at RT. The dried samples were coated with gold/Palladium using Gold/Palladium Target 57 mm diameter x 0.1 mm thick (Gold/Palladium Target: Electron Microscopy Sciences, Hatfield, PA) with research grade argon gas. SEM were operated at 15 kV with top view. *Finally*, Co-imaging of confocal microscopy with SEM were used to analyze the intracellular trafficking of YOYO-IL-4pDNA-NPs in the MDM.

### Enzyme-Linked Immunosorbent Assay (ELISA)

ELISA was performed on culture media to determine the secretion of cytokines using ELISA Kits purchased from eBioscience (San Diego, CA). The supernatant was collected at 1, 3, and 5 days post transfection and were analyzed according to the manufacturer’s protocol.

### Inflammatory Mouse Model and Treatment

The mouse model of inflammation was established with intraperitoneal injection of lipopolysaccharide (LPS; 100 μg) in Balb/C mice. The pre-treated group was administered 100 μL of mouse IL-4pDNA-NPs through the tail vein 24 hours before LPS injection. The post-treated group was given IL-4pDNA-NPs 24 hours after LPS injection. LPS only and normal mouse served as the control. All experiments in Balb/C mice were carried out in strict accordance with the recommendations in the Guide for the Care and Use of Laboratory Animals of the National Research Council. The protocols were approved by the Texas Tech University Health Sciences Center El Paso (TTUHSC EP) Institutional Animal Care and Use Committee. All mice were purchased from Jackson Laboratories (Bar Harbor, ME) at 4 weeks and were raised and maintained by the Laboratory Animal Resource Center at TTUHSC.

### Histology and Immunofluorescence

Mice were humanely euthanized at day 7 and the spleen was collected and the sections were prepared (5 µm thick) for staining. The immunostaining was performed with antibodies of IL-4 (1:200; Novus Biologicals, Littleton, CO), iNOS (1:200; Abcam, Cambridge, United Kingdom), Arginase-1 (1:200, Thermo Fisher Scientific, Waltham, MA), CD11b (1:200; abcam, Cambridge, Ma, USA). Secondary antibodies used were goat anti rat Alexa Fluor 488 and goat anti rabbit Alexa Fluor 546 (Thermo Fisher Scientific, Waltham, MA). The nuclei were stained with DAPI. Immunofluorescence images were observed under a microscope (Nikon Instruments Inc., Melville, NY).

### Flow Cytometry

MDM cells treated with human IL-4pDNA/GFP-NPs were scraped and fixed in 2% paraformaldehyde (Sigma-Aldrich, St Louis, MO) and analyzed on a BD FACS Canto II flow cytometer (BD, Franklin Lakes, NJ). Data on 5 x 10^5^ cells were collected and the percentage of GFP positive cells and fluorescence intensity between conditions were analyzed with FlowJo software (FlowJo, LLC, Ashland, OR).

For *in vivo* studies, the spleen was collected from mice and was broken down through a 40 μM strainer. Cells were centrifuged for 5 minutes at 1200 rpm, and then the supernatant was discarded. Cells were resuspended in 5 mL ACK buffer and were incubated at room temperature for 5 minutes. 5 mL’s of DPBS was added to stop the reaction, and the cell suspension was strained through a 40 μM strainer. The solution was centrifuged for 5 minutes at 1200 rpm, then the cells were resuspended in blocking buffer (3% BSA in PBS) and counted. 1 x 10^6^ cells were blocked for 1 hour per condition, then samples were incubated for 30 minutes with CD45 PE-Cy7, CD11b Alexa Fluor 700, iNOS APC, and Arginase 1 PE (Thermo Fisher Scientific, Waltham, MA) at 4°C. Samples were then washed 3 times in wash buffer (1X BSA in PBS) and fixed (1X BSA, 2% PFA, in PBS). Samples were immediately analyzed on a Gallios Flow Cytometer (Beckman Coulter, Brea, CA). Data on 5 x 10^5^ cells were collected and analyzed with FlowJo software. 2 – 3 mice were analyzed per condition, and the experiment was repeated in triplicate.

### Statistical Analysis

SPSS software was used for all statistical analyses and all data are presented as the mean ± standard error of the mean (SEM). In all groups, P values <0.05 were considered statistically significant. For comparison of groups, we used one-way analysis of variance (ANOVA) Student’s t-test. All experiments were repeated in triplicate.

## Results

### IL-4pDNA-NPs Characterization

**Figure 1:**
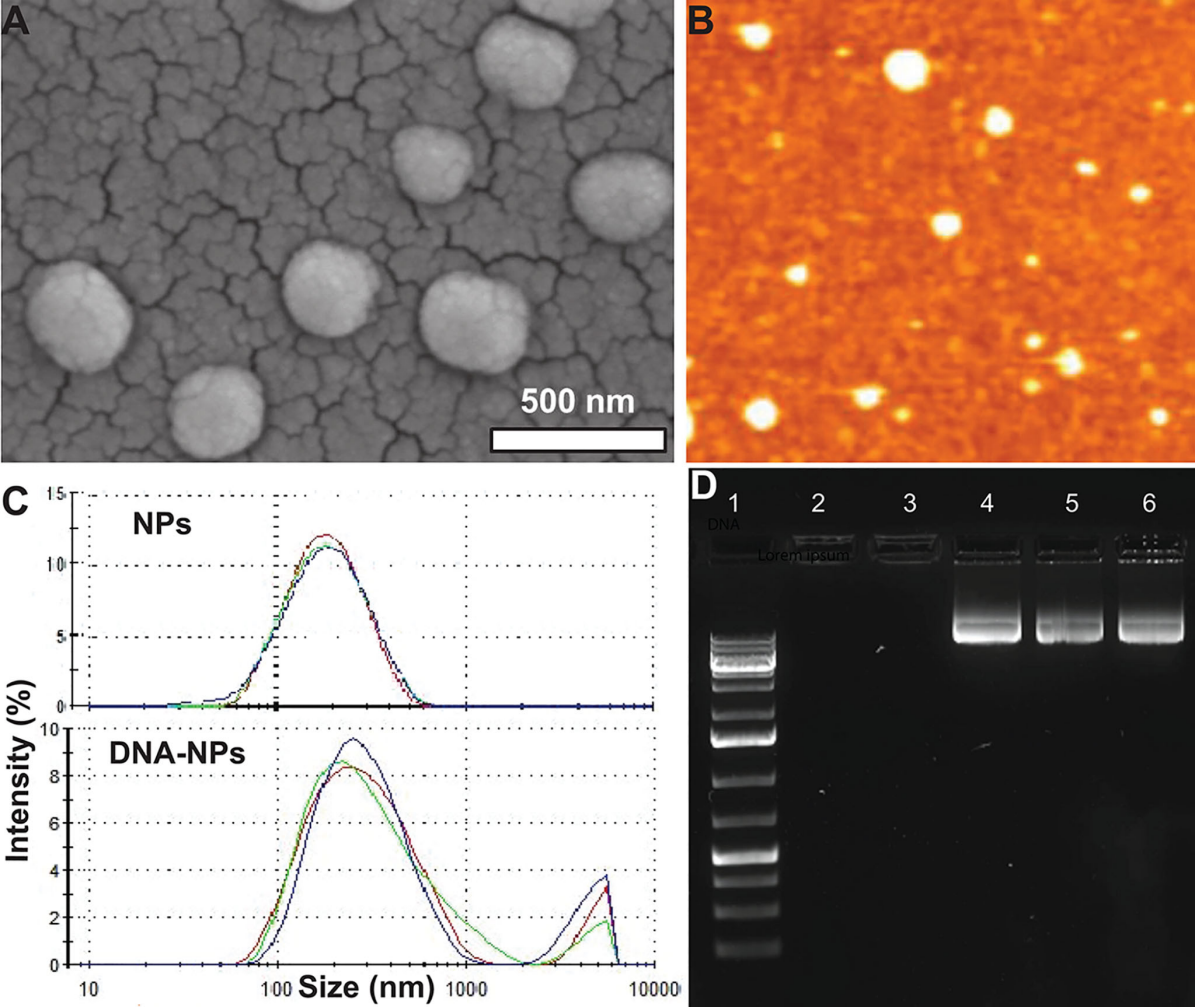
Characterization of IL-4pDNA-NPs. **(A)** SEM and **(B)** AFM images show that NPs are spherically shaped, smooth and are monodispersed. **(C)** Size distribution of unloaded NPs (NPs) and human IL-4pDNA loaded NPs (IL-4pDNA-NPs). **(D)** Encapsulation of IL-4pDNA by NPs at various N/P ratios was detected by Agarose gel electrophoresis. Lane 1 is DNA ladder as size marker. Lanes 2 - 6 show IL-4pDNA-NPs at N/P ratios of 4, 2.6, 2, 1.8, and 1.6. No bands were detected at N/P ratios of 4 and 2.6, indicating full encapsulation. Unencapsulated IL-4pDNA bands were obtained from N/P ratios below 2, demonstrating overloading of pDNA into NPs.

The cationic branched PEI lipid was first synthesized by reacting low molecular weight, branched PEI (MW = 800 g/mol) with GHE (MW = 298 g/mol) at a molar ratio of 1:8 respectively. This ratio was experimentally determined in our previous work to enhance transfection efficiency while decreasing cytotoxicity (29). A nanoformulation was made with this cationic branched PEI lipid shell and PLGA core (NPs) through a modified solvent evaporation procedure. The polar hydrophilic headgroup of highly positive cationic lipids enable the condensation of negative charged IL-4 pDNA. A plasmid containing full length IL-4 pDNA tagged with GFP was loaded into NPs (IL-4pDNA/GFP-NPs). SEM and AFM assays (**Figures 1A, B**) indicated that these NPs had a smooth surface, are spherically shaped, and are monodispersed, suggesting a successful nanoformulation. The particle size, polydispersity index (PDI) and surface charge were also characterized by a zetasizer. IL-4 pDNA/GFP-NPs at an N/P ratio of 1:10 showed a mean diameter of 282.3 ± 6.4 nm (**Figure 1C**). Note that the particle size increased with loading the IL-4 pDNA. This is consistent with the data presented in the SEM images in **Figure 1A**. The PDI, a measure of the broadness of the molecular weight distribution, is 0.481 ± 0.009. PDI values under 0.5 indicates that the NPs are monodispersed in the solution (32). Finally, the zeta potential, a measure of stability, showed IL-4pDNA-NPs were +36 ± 7.13 mV. Values larger than ±35 mV suggest the NPs are stable (33, 34), while the positive charge represents the cationic nature of the PEI lipid (35, 36). Together, these data indicate our newly synthesized NPs are cationic and stable in a colloidal suspension, and are homogeneous in size and shape. A facile method to synthesize cationic lipids using low molecular weight PEI and NPs successfully delivered IL-4 pDNA (29) to enhance the transfection. Furthermore, the stability of NPs were up to 3 months (data not shown).

To determine the encapsulation capacity, NPs were loaded with IL-4pDNA at various molar ratios of PEI nitrogen’s (N) to DNA phosphates (P) up to N/P=10. Encapsulation capacity of IL-4pDNA/GFP-NPs was determined by agarose gel electrophoresis at N/P ratios of 4, 2.6, 2, 1.8, and 1.6 (**Figure 1D** *, Lane 2-6*). DNA ladder was used as the size marker (**Figure 1D** *, Lane 1*). The results indicated that IL-4pDNA-NPs at N/P ratios of 4 and 2.6 were fully encapsulated after 30 minutes of incubation. By increasing IL-4pDNA concentration to N/P ratios of 2, 1.8, and 1.6, a positive band was detected on the gel that indicates the over-loading of unencapsulated IL-4pDNA. Further, the loading capacity was confirmed using unloaded IL-4pDNA as control to run the gel (**Supplementary Figure 1**)

### MDM Transfection With IL-4pDNA-NPs

**Figure 2:**
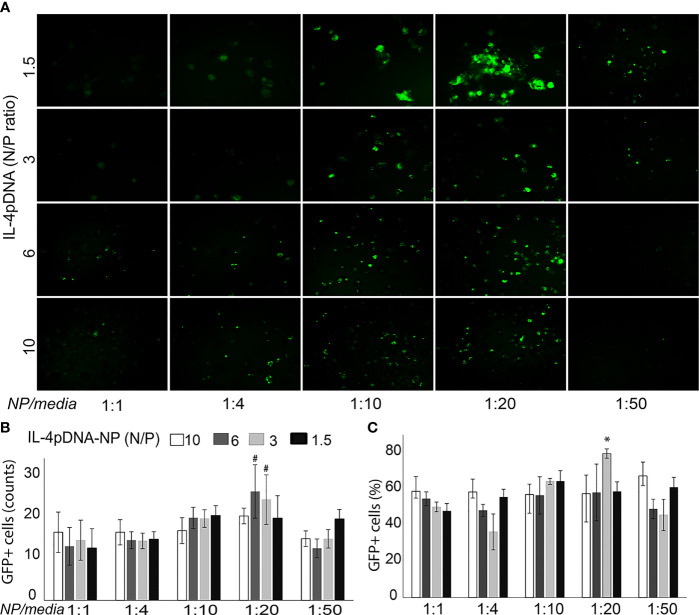
Transfection of MDM by IL-4pDNA-NPs. **(A)** Fluorescence microscopy imaging of GFP+ MDM cells illustrates the transfection of IL-4pDNA/GFP-NPs at various N/P ratios of IL-4pDNA with different NP concentrations (NPs to culture media volume ratios). **(B)** The absolute numbers of GFP+ MDM were counted and the best transfection was obtained with IL-4pDNA/GFP-NPs at N/P ratios of 6 and 3 and NPs to culture media volume ratio of 1:20. **(C)** The percentages of GFP+ MDM were calculated and indicate the highest transfection occurred with 1.5 ng/ml of IL-4pDNA/GFP-NPs at a NPs to culture media ratio of 1:20. Error bars represent the standard error of the mean (s.e.m.). Note that all experiments were done in triplicate. *P<0.05 compared to all conditions; #*P<0.05* represented N/P ratio at 3 or 6 to compare all conditions except between the N/P ratio at 6 and 3 in 1:20.

Transfection of MDMs with IL-4pDNA/GFP-NPs was evaluated by examining GFP expression (**Figure 2A** *, green*). MDMs were cultured up to 7 days with 1000 units of human MCSF, and were allowed to differentiate into macrophages. MDMs were then treated with IL-4pDNA/GFP-NPs at N/P ratios of 10, 6, 3, and 1.5 with NPs to media ratios of 1:1, 1:4, 1:10, 1:20, and 1:50. GFP+ transfected MDM were seen in all conditions (**Figure 2A**). An increase of GFP+ MDM was seen with a NPs to media ratio from 1:1 up to 1:20. Interestingly, the transfection efficiency decreased with a NPs to media ratio of 1:50. The absolute numbers of GFP+ MDM were counted (**Figure 2B**), indicating successful transfection in all conditions. The highest GFP+ MDM counts occurred with transfection of IL-4pDNA/GFP-NPs at N/P ratios of 6 and 3 with a NPs to media ratio of 1:20. The percentage of GFP+ MDM was significantly increased in IL-4pDNA/GFP-NPs at N/P ratio 3 with NPs to media ratio of 1:20 (**Figure 2C**). Flow cytometry confirmed the increases of GFP+ MDM following treatment with IL-4pDNA-NPs at ratios of 1:1, 1:4, 1:10, 1:20 and 1:50 at day 5 (**Supplementary Figure 2**). The merged flow cytometry histograms illustrate there is GFP+ expression in MDM in all conditions.

### Real-Time Live Cell Tracking of Intracellular IL-4 pDNA/GFP-NPs and Nuclear Entry

**Figure 3:**
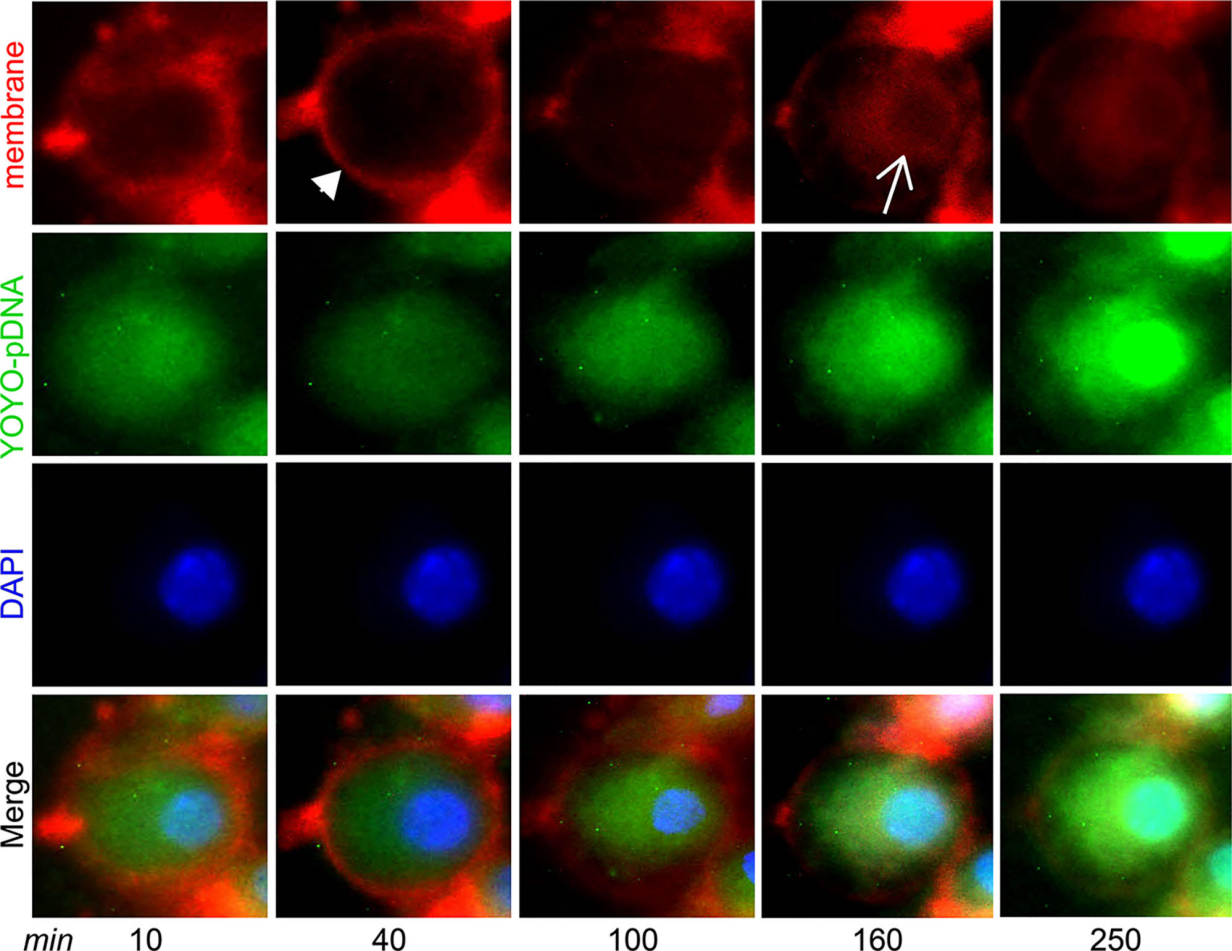
YOYO-IL-4pDNA/NPs cross the cell and nuclear membranes to enter the nucleus. Live cell imaging was used to assess cytoplasmic transportation and the nuclear entrance of YOYO-IL-4pDNA-NPs. The cultures were treated with YOYO-IL-4pDNA-NPs **(green)** and the cell membranes were labeled with CellBrite Fix555 **(red)**. The nuclei were labeled with DAPI **(blue)**. The images were initially taken at 10 minutes and automatically scanned every 30 minutes under live cell fluorescence microscopy. YOYO-IL-4pDNA were seen to cross the cell membrane **(red, arrow head)** at 30 minutes. Red CellBrite Fix555 labeled the nuclear envelop **(red, arrow)** as seen at 160 minutes, showing YOYO-IL-4pDNA entered the nucleus. After 250 minutes, YOYO-IL-4pDNA was highly accumulated within the nucleus **(bright green)**, suggesting the cytoplasmic transportation of YOYO-IL-4pDNA into the nucleus.

Intracellular trafficking of IL-4pDNA was performed by a real-time cell imaging assay. IL-4pDNA was labeled with the green dye YOYO-1 iodide (YOYO-IL-4pDNA), then loaded into NPs (YOYO-IL-4pDNA-NPs). The membranes of the cells were labeled with red dye CellBrite Fix555 and the nucleus was tracked by DAPI. Triple labeling of YOYO-IL-4pDNA-NPs, the cell membranes and the nucleus was initially imaged at 10 minutes followed by automatic scanning every 30 minutes. Live cell images showed that YOYO-IL-4pDNA-NPs (**Figure 3** *, green*) crossed the cell membranes (**Figure 3** *, red, narrow head*) and entered the cytoplasm at 40 minutes. The levels of YOYO-IL-4pDNA-NPs consistently increased inside of cells up to 250 minutes. The nuclear envelop (**Figure 3** *, red, arrow*) was labeled at 160 minutes after exposure to CellBrite Fix555, while “green” YOYO-IL-4pDNA traversed across the “red” nuclear membranes to enter into the “blue” nucleus. Importantly, consistent nuclear accumulation of YOYO-IL-4pDNA was obtained from 160 to 250 minutes (**Figure 3**). We observed YOYO-IL-4pDNA-NPs were quickly taken up by macrophages that are actively transported throughout the cytoplasm, allowing nuclear accumulation of YOYO-IL-4pDNA for effective delivery.

### SEM and Fluorescence Microscopy Show the Intracellular Transportation of IL-4 pDNA/GFP-NPs

IL-4pDNA escape from lysosomes to enter the nucleus is key for gene delivery and plasmid transfection. Lysosomes are cellular organelles responsible for digestion and removal of foreign objects in cells. As professional phagocytes, lysosomes in MDM are largely activated during uptake of “foreign” IL-4pDNA-NPs (**Supplementary Figure 3A**). We detected the lysosomal responses to intracellular IL-4pDNA-NPs in mouse bone marrow-derived macrophages (BMM) at different time-points (**Supplementary Figure 3B**). Maximal increase of lysosomes in BMM was detected at 1 day of IL-4pDNA-NPs treatment (**Supplementary Figure 3C**). This presents a major challenge to develop a carrier that can escape from lysosomes and traverse to the nucleus.

**Figure 4:**
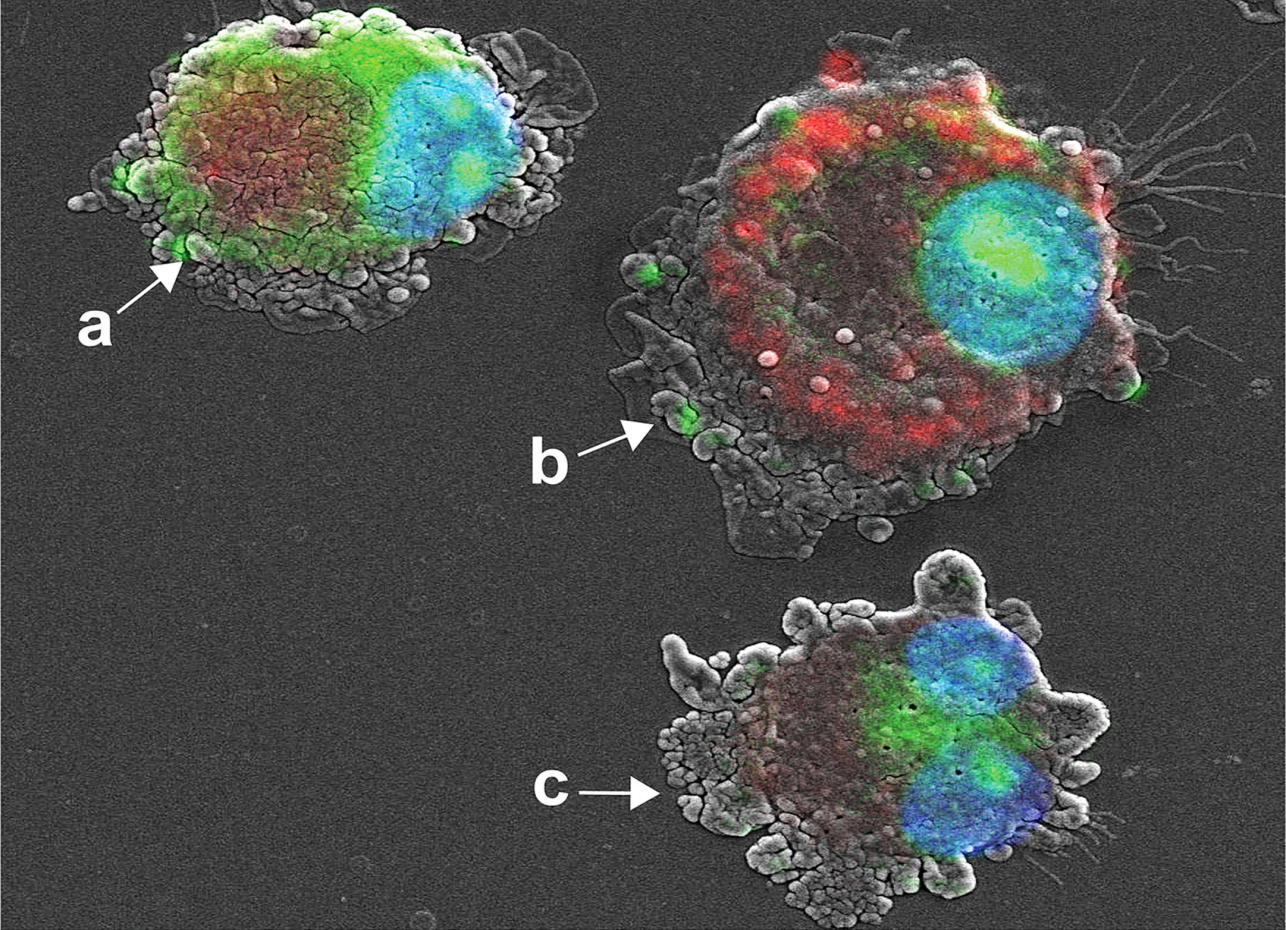
Cytoplasmic transportation of YOYO-IL-4pDNA/NPs. Confocal microscopy and SEM images were taken of MDM transfected with YOYO-IL-4pDNA-NPs for co-imaging assay. Co-imaging of microstructural evaluation was used to determine the intracellular YOYO-IL-4pDNA-NPs escaping from LysoTracker labeled lysosomes (red) to enter the nucleus (blue). Co-imaging showed co-localization of YOYO-IL-4pDNA-NPs **(a, green)** with lysosomes **(a, red)** by MDM. YOYO-IL-4pDNA-NPs escaped from lysosomes **(b, red)**, resulting in the separation of “red” lysosomes and “green” YOYO-IL-4pDNA. The escaped YOYO-IL-4pDNA-NPs **(c, green)** from lysosomes were transported into the nucleus (blue).

We developed a co-imaging assay using fluorescence microscopy and SEM for microstructural trafficking of YOYO-IL-4pDNA-NPs. MDM were treated with YOYO-IL-4pDNA (green) and lysosomes were labeled with LysoTracker (red). To locate the nucleus, DAPI (blue) staining was applied. The microstructures of MDM were co-imaged to reveal the escape of YOYO-IL-4pDNA from lysosomes for nuclear ingress. The image was taken for 2 hours after YOYO-IL-4pDNA-NPs treatment. Three phases of cytoplasmic transportation (**Figure 4**) show the uptake of YOYO-IL-4pDNA-NPs (**Figure 4A** *, green*) by MDM, the separation of “green” YOYO-IL-4pDNA-NPs from “red” lysosomes (**Figure 4-b**) and the transportation of YOYO-IL-4pDNA integration into the nucleus (**Figure 4-c**). Escape of YOYO-IL-4pDNA-NPs from lysosomes showed the effective delivery of pDNA by NPs. Nuclear ingress of YOYO-IL-4pDNA within 2 hours indicated that YOYO-IL-4pDNA-NPs can effectively cross the cytoplasmic membranes and the nuclear envelop to deliver pDNA for MDM transfection.

**Figure 5:**
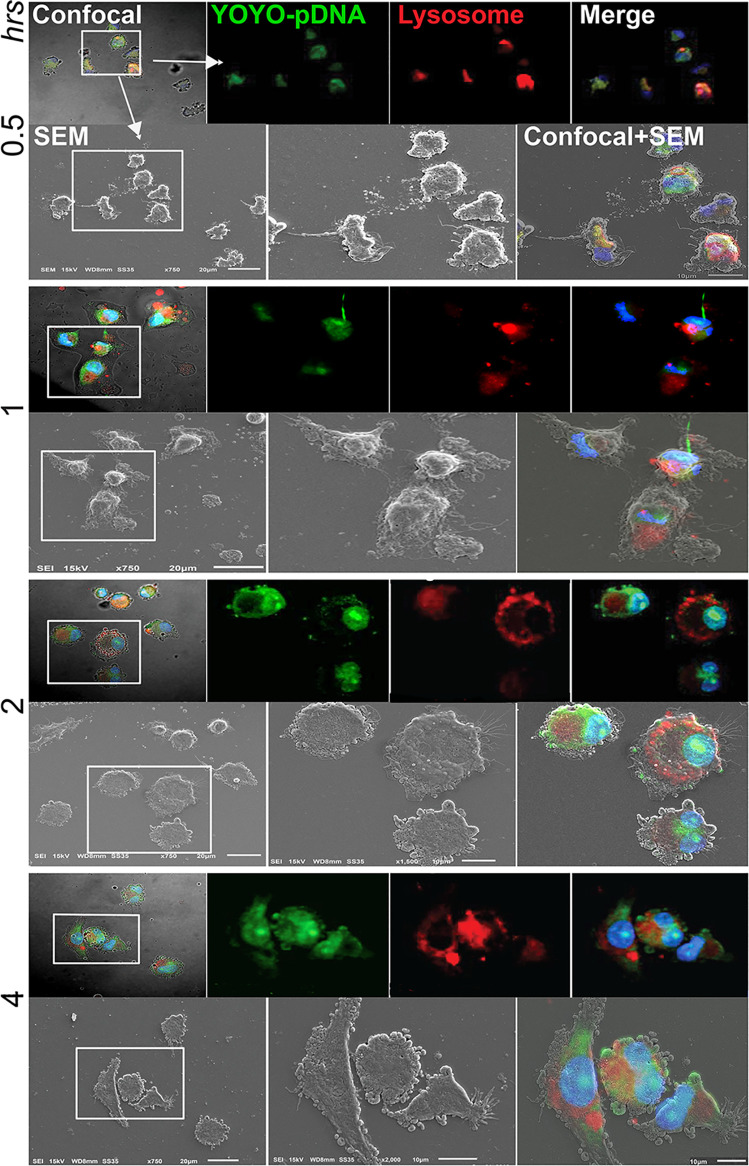
Intracellular trafficking and nuclear ingress of YOYO-IL-4pDNA/NPs. Co-imaging of confocal microscopy and SEM were taken of MDM transfected with YOYO-IL-4pDNA-NPs at 0.5, 1, 2 and 4 hours. Lysosomes were labeled with LysoTracker (red) and nuclei were labeled with DAPI (blue). For each time point, the confocal (upper panel) images were taken before scanning with the SEM (lower panel). Co-images are shown in the lower panel (confocal + SEM). YOYO-IL-4pDNA-NPs were internalized in MDM cells at 0.5 hours. The escaped YOYO-IL-4pDNA-NPs from lysosomes were seen at 1 and 2 hours, and condensed in the nucleus by 2 hours post transfection. By 4 hours, greater cytoplasmic levels of YOYO-IL-4pDNA-NPs were detected again, indicating continual intracellular accumulation within the cell body.

Time-dependent intracellular trafficking of IL-4 pDNA/GFP-NPs in MDM was assessed by the co-imaging assay (**Figure 5**). MDM were treated with YOYO-IL-4pDNA-NPs for 0.5, 1, 2, 4 hours. Lysosomes and nuclei were labeled with LysoTracker and DAPI, respectively (**Figure 2C**). Co-images from the SEM and confocal microscopes revealed YOYO-IL-4pDNA (**Figure 5**, *green*) were taken in by MDM at 30 minutes, and were immediately captured within lysosomes (**Figure 5**, *red*) indicated by “yellow”. Separation of “green” YOYO-IL-4pDNA from “red” lysosomes occurred at 1 and 2 hours, showing successful escape of YOYO-IL-4pDNA-NPs from lysosomes. YOYO-IL-4pDNA traversed into the nucleus within 2 hours where it remained for the next 2 hours. At 4 hours, MDM continued to uptake YOYO-IL-4pDNA-NPs within the cytoplasm. These results strongly indicate that PLGA/PEI NPs facilitated MDM uptake to continually maintain the intracellular levels of IL-4pDNA for effective transfection.

### Analysis of Transfection Stability in MDM

**Figure 6:**
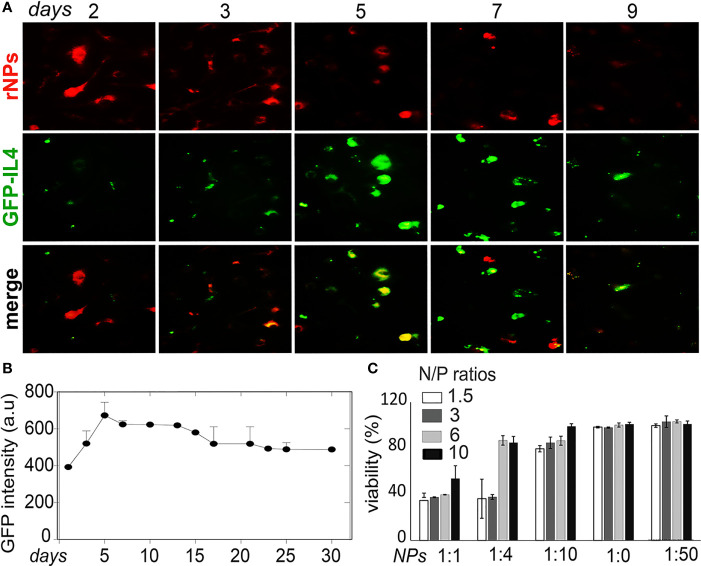
Stability of IL-4pDNA-NPs transfection. **(A)** NPs were labeled with red dye Rhodamine (rNPs) and loaded with IL-4-pDNA/GFP (IL-4pDNA/GFP-rNPs). MDM were treated with IL-4pDNA/GFP-rNPs for 2, 3, 5, 7 and 9 days to evaluate the correlation of internalized rNPs and GFP expression in MDM. The greatest intracellular rNPs accumulation (A, red) was seen at day 2, followed by gradual reduction over 9 days. A peak level of GFP+ intensity (A, green) was obtained at day 5 with continual expression up to 9 days. **(B)** GFP expression was detected over the next 30 days. **(C)** Specific toxicity of different ratios of NPs to culture media for various concentrations of IL-4pDNA was evaluated by a viability assay. No toxicity was observed at NPs to media ratios of 1:20 and 1:50. Error bars represent the standard error of the mean (s.e.m.).

NPs were labeled with red dye Rhodamine 6G (rNPs) and loaded with GFP-tagged IL-4pDNA (IL-4pDNA/GFP-rNPs). The intracellular red fluorescence signals were imaged to determine the uptake and the degradation of IL-4pDNA/GFP-rNPs in MDM. GFP expression (green) in MDM revealed successful transfection. IL-4pDNA/GFP-rNPs were internalized into MDM within 1 day and gradually decreased from days 2 to 9 (**Figure 6A** *, red)*. IL-4/GFP expression continually increased from day 2 with the highest GFP expression seen on day 5 (**Figure 6A** *, green*). IL-4/GFP expression subsequently decreased at days 6 to 9. To identify the stability of IL-4/GFP expression, GFP fluorescence intensity was examined in a time-period of 30 days. Peak GFP intensity occurred on day 5 that decreased by day 10, with sustained levels up to 30 days (**Figure 6B** and **Supplementary Figure 4A**).

Cytotoxicity is a major concern when developing a nanoparticle-based gene delivery system. Specific toxicity of different ratios of NPs to culture media for various concentrations of IL-4pDNA was evaluated to assess the safety of IL-4pDNA/GFP-NPs. MDM were treated with 1, 1.5, 3, and 6 ng/ml of IL-4pDNA-NPs at NPs to culture media ratios of 1:1, 1:4, 1:10, 1:20, and 1:50. The viability assay was performed on day 5 (**Figure 6C**) post transfection. The cultures treated with IL-4pDNA/GFP-NPs at 1:20 and 1:50 demonstrated greater viability. In contrast, higher concentrations of IL-4pDNA/GFP-NPs treated to cultures displayed increased toxicity at NPs to media ratios of 1:10, 1:4 and 1:1. The data suggest higher concentrations of IL-4pDNA/GFP-NPs were toxic to the cells.

### Exogenous IL-4 Production Induced MDM Responses

**Figure 7:**
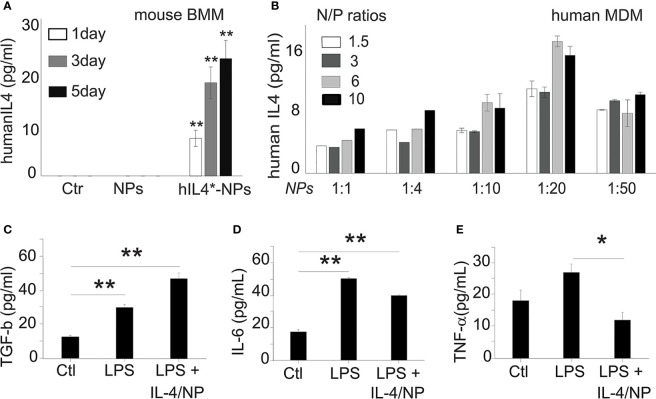
Exogenous IL-4 production induces MDM responses. **(A)** Mouse bone marrow derived macrophages (BMM) were used to determine human IL-4pDNA transfection and production. An ELISA assay with human specific antibodies showed human IL-4 secretion from mouse BMM transfected with IL-4pDNA-NPs. The human IL-4 expression in mouse BMM were increased from day 1 to day 5 (**p<0.01 hIL4-NPs v.s Ctr and NPs). No human IL-4 was detected in mouse BMM in normal and NPs control groups. These results indicate the successful expression of exogenous IL-4 production in macrophages. **(B)** The secretion of IL-4 from MDM was quantified with different IL-4pDNA-NP concentrations at various NP/media ratios. IL-4 protein was detected in the media for all conditions, while the highest expression was obtained in MDM treated with IL-4pDNA-NPs at N/P ratios of 6 and under, and a NPs to media volume ratio at 1:20. Exogenous IL-4 was produced from IL-4pDNA-NP transfected MDM. Polarizing M1 macrophages into M2 by exogenous IL-4 was then investigated in LPS induced inflammatory M1 MDM with IL-4pDNA-NPs (LPS+IL4/NPs). Untreated MDM (Ctr) and LPS alone (LPS) served as the controls. ELISA was used to detect the levels of TGF-β, IL-6 and TNF-α. **(C)** TGF-β, an anti-inflammatory cytokine and a marker of M2 macrophages, is significantly increased in IL-4pDNA-NP treated cultures compared to MDM and LPS-inflammatory MDM (*p< 0.01). Inflammatory M1 markers of IL-6 **(D)** and TNF-α **(E)** were increased in LPS-inflammatory MDM. However, IL-4pDNA-NPs treatment decreased the secretion of IL-6 and TNF-α (*p < 0.01). MDM responded to the production of exogenous IL-4 and polarized M1 into M2. Significance was evaluated with a one-way ANOVA followed by Tukey’s *post-hoc* analysis. Error bars represent s.e.m. *p < 0.05, **p < 0.01.

Next, we studied how IL-4pDNA-NP transfection manipulates macrophage immunological functions. Because macrophages can produce IL-4, we verified the production of induced human IL-4 in IL-4pDNA/GFP-NP transfected mouse bone marrow derived macrophages (BMM). The secretion of human IL-4 from mouse BMM was evaluated by ELISA using an antibody specific to human IL-4. BMM and NPs without pDNA treatment served as controls. The culture medium from day 1, 3 and 5 indicated significant production of human IL-4 from transfected mouse BMM (**Figure 7A**). A large increase of human IL-4 was obtained at day 3 and 5 post transfection. A series of human IL-4pDNA-NPs were treated to human MDM. The levels of IL-4 in the culture media (**Figure 7B**) were analyzed by ELISA at day 5. The highest level of IL-4 was obtained at an N/P ratio of 6 with NPs to media ratio of 1:20 (*p<0.01* to all conditions except N/P ratio of 10 with NPs to media ratio of 1:20). IL-4pDNA-NPs can achieve gene delivery in macrophages.

The regulatory role of IL-4pDNA-NPs transfection was investigated by examining LPS-induced inflammatory MDM (M1) polarization into immunosuppressive M2. The pro-inflammatory cytokines TNF-α and IL-6, and the anti-inflammatory cytokine TGF-β were used to characterized M1/M2 polarization. Non-treated MDM served as the control. Examination of the M2 marker TGF-β was significantly increased in IL-4pDNA-NP treated inflammatory M1 MDM cells when compared to the control and LPS-induced M1 cells (**Figure 7C**). This suggests IL-4pDNA-NPs were capable of repolarizing inflammatory macrophages by overexpression of anti-inflammatory cytokine TGF-β. LPS treated inflammatory M1 MDM showed an increase in the M1 markers IL-6 (**Figure 7D**) and TNF-α (**Figure 7E**). With IL-4-pDNA-NPs treatment, there was a 23% reduction of IL-6 production compared and a significant reduction in the level of TNF-α to LPS alone (p < 0.01). Our data suggests IL-4pDNA-NPs upregulated the M2 cytokine TGF-β and downregulated LPS-induced inflammatory cytokines TNF-α and IL-6, leading to functional regulation of M1/M2 cytokine signaling pathways for MDM repolarization.

We further characterized IL-4-pDNA-NPs inhibition of the inflammatory marker iNOS in MDM by flow cytometry. MDM were either pre-treated with IL-4pDNA-NPs followed by exposure to LPS (pre-NPs+LPS), or post-treated (NPs+LPS) with IL-4pDNA-NPs 1 day after LPS exposure. MDM were exposed to LPS to induce the overexpression of iNOS. Flow cytometry results revealed a decrease of iNOS+ MDM in pre- and post-IL-4pDNA-NP treated cultures (**Supplementary Figure 4B** ***)***. There was significant inhibition of iNOS in IL-4pDNA-NPs pre- and post-treated inflammatory MDM (**Supplementary Figure 4C**), suggesting IL-4pDNA-NPs inhibited LPS-induced overexpression of iNOS in MDM.

### IL-4pDNA-NPs Transfection *In Vivo*

The *in vivo* delivery effectiveness of IL-4pDNA-NPs was tested in Balb/C mouse. All experiments in Balb/C mice were carried out in strict accordance with the recommendations in the Guide for the Care and Use of Laboratory Animals of the National Research Council. The protocols were approved by the Texas Tech University Health Sciences Center El Paso (TTUHSC EP) Institutional Animal Care and Use Committee. Mice were administered IL-4pDNA/GFP-rNPs *via* the tail vein at a NP to blood volume ratio of 1:20. The spleens were collected at day 5 and fresh spleen sections were scanned by fluorescence microscopy. The images suggest that IL-4/GFP expression (**Supplementary Figure 5** *, green*) co-localized with the cells that internalized IL-4pDNA/GFP-rNPs (**Supplementary Figure 5** *, red*). Specifically, the “green” GFP+ and “red” rNPs were present in the border of the marginal zone and outer periarteriolar lymphatic sheaths of the spleen (**Supplementary Figure 5** *, merge*). This indicates that splenic macrophages can uptake IL-4pDNA/GFP-rNPs *in vivo*.

**Figure 8:**
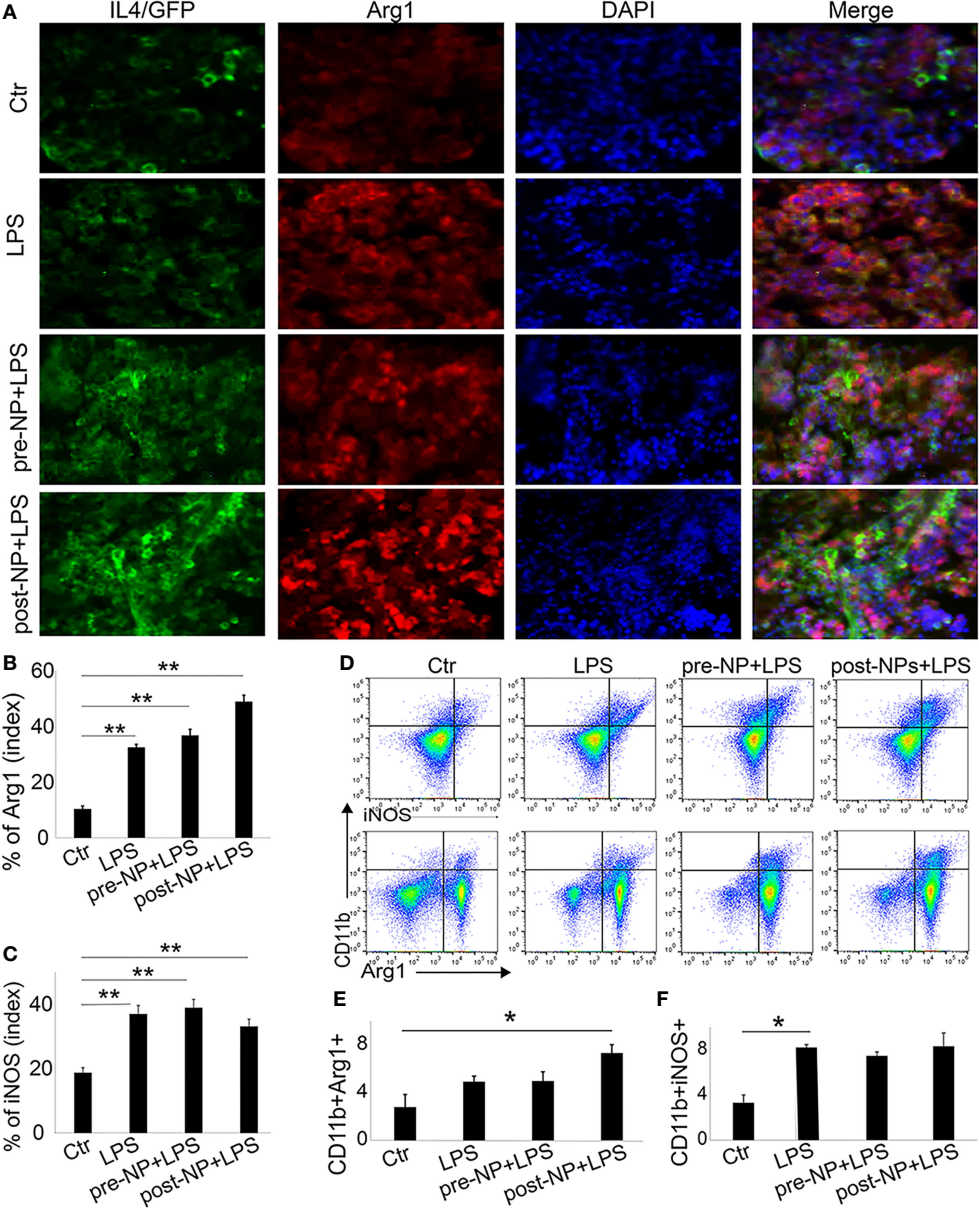
Spleen macrophages respond to IL-4pDNA-NPs in a mouse model of inflammation. **(A)** A histological assay was performed in an inflammation mouse model that was treated with IL-4pDNA-NPs 24 hours before (pre-NP+LPS) or after (post-NP+LPS) LPS induction. LPS-inflammation mice (LPS) and normal animals (Ctr) served as controls. The spleen sections from all experimental mice were stained with antibodies to IL-4 (**A**, green) and the M2 marker arginase 1 (Arg 1) (**A**, red). Microscopy images showed that overexpression of IL-4+ in IL-4pDNA-NP treated mice correlated with the increases of Arg-1 in spleen sections. Quantification of Arg-1 positive staining **(B)** showed a significant increase in both IL-4pDNA-NP pre-and post-treated LPS mice when compared to untreated inflammatory mice. This suggests M2 polarization by IL-4pDNA-NPs treatment. **(C)** The inflammatory M1 marker iNOS was overexpressed in LPS-mice. IL-4pDNA-NPs treatment to LPS-mice inhibited iNOS expression. **(D)** Representative flow cytometry plots illustrated the typical gating strategy to identify CD11b+Arg-1+ and CD11b+iNOS+ macrophages in spleen. **(E)** The percentages of CD11b+/Arg-1+ macrophages showed a significant increase in IL-4pDNA-NP treated LPS mice. **(F)** LPS enhanced CD11b+/iNOS+ macrophages in spleen. No significant changes were seen in LPS-mice with or without IL-4pDNA-NPs treatment. Significance was evaluated with a one-way ANOVA followed by Tukey’s *post-hoc* analysis. Error bars represent s.e.m. *p < 0.05, **p < 0.01.

We further investigated the anti-inflammatory role of IL-4pDNA/GFP-rNPs in an LPS-induced inflammation mouse model. For comparison, pre-treatment mice were given IL-4pDNA-NPs 24 hours before LPS injection (pre-NP+LPS). The post-treatment group received IL-4pDNA-NPs 24 hours after LPS-induction (post-NP+LPS). Normal mice and LPS only mice served as the control (Ctl and LPS, respectively). The spleen sections were collected at day 7 post LPS-induction. The spleen sections were stained with antibodies against arginase-1 (Arg1, M2 marker) and iNOS (M1 marker). IL-4 staining was used to evaluate IL-4pDNA-NPs transfection in mouse spleen. Immunofluorescence images revealed an overexpression of IL-4 (**Figure 8A** *, green*) in mice treated with IL-4pDNA-NPs, suggesting successful transfection of IL-4 *in vivo*. The M2 marker Arg-1 (**Figure 8A** *, red*) was largely increased in both pre-NP+LPS and post-NP+LPS groups compared to the control and LPS only groups. The greatest Arg-1 expression was seen in post-NP+LPS mice that also showed the highest IL-4 positive staining. Quantitative imaging confirmed the statistical increase of Arg-1 in LPS, pre-NP+LPS and post-NP+LPS groups compared to the control (**Figure 8B**). Immunostaining for the M1 marker iNOS in spleen sections showed increases in all conditions compared to the control (**Figure 8C**). Importantly, IL-4pDNA-NPs treatment decreased the LPS-induced iNOS over-expression. These results suggest that administration of IL-4pDNA-NPs can polarize M1 macrophages towards an M2 phenotype against inflammation *in vivo.*

To confirm these results, fresh spleen cells were isolated and analyzed by flow cytometry. The splenic macrophages were gated on CD45+ and CD11b+. The CD11b+/Arg-1+ and CD11b+/iNOS+ macrophages (**Figure 8D**) were analyzed to reveal M2 and M1 subpopulations. Notably, a significant increase of CD11b+/Arg-1+ M2 macrophages was obtained in post-NPs+LPS mice when compared to control (**Figure 8E**). This result indicated IL-4pDNA-NPs polarized macrophages towards the M2 phenotype after inflammation. The increase of iNOS expression was seen in all conditions when compared to the control (**Figure 8F**) similar to the spleen sections with immunostaining. Together, these data further support the evidence of M2 polarization by IL-4pDNA-NPs after inflammation *in vivo*.

## Discussion

We synthesized cationic PEI lipids using branched low molecular weight PEI (29) to develop core-shell NPs for gene editing of non-dividing cells, such as macrophages. Human MDM were successfully transfected with full length IL-4 pDNA/GFP, as a proof-of-concept, that was delivered by the newly synthesized NPs. IL-4pDNA-NPs were delivered as a genetic regulator into human primary blood monocyte derived macrophages, to produce IL-4 to stimulate anti-inflammatory genes and to inhibit pro-inflammatory cytokines for therapeutic immune rebalancing.

Macrophages play a major role in both activating and attenuating the immune system, and are therefore one of the prime targets for immunotherapies (37). However, transfecting macrophages is difficult, therefore developing new gene delivery technologies is of the upmost importance (38–42). We developed cationic NPs capable of transfecting human MDM, mouse BMM, and mouse splenocytes. Importantly, IL-4pDNA-NPs were capable of repolarizing M1 macrophages into M2 in human MDM, mouse BMM, and in a mouse model. Therefore, the delivery of IL-4 pDNA-NPs into macrophages, and the subsequent induction of IL-4 expression and M2 repolarization, provides a new gene therapy solution for the treatment of various diseases where the need of attenuating an over reactive immune response is desired.

Nanotechnology is emerging as a new gene-based therapy for the treatment of many diseases. However, it has several drawbacks that prevent its use in the clinical setting. The limiting factors of this technology include low transfection rates and high cytotoxicity. PEI NPs, the gold standard for gene transfection, has shown promising results, but how well it works is dependent on the molecular weight of PEI (43–45). PEI with an average molecular weight of 25,000 g/mol shows high transfection efficiency with high cytotoxicity. Low molecular weight PEI and branched PEI’s of both high and low molecular weight have also been investigated, and show low transfection rates (46). To circumvent these issues, we found low molecular weight, branched, PEI, when reacted with GHE, produced a highly stable cationic lipid. This lipid allows for a highly efficient capture and delivery of pDNA to the cell. When paired with PLGA and DMG-PEG through a modified solvent evaporation procedure, we were able to form core-shell NPs. In addition, synthesizing PEI lipids and NPs require stable temperature and are sensitive to sonication power and time. This core-shell NP allows the pDNA to remain stable in the tissue longer, and it improves cellular uptake and transport while reducing cytotoxic effects (47).

Non-dividing cells and primary macrophages are difficult to transfect (48, 49). They show poor DNA internalization, activate immune pathways in response to foreign objects, and do not divide, thereby preventing nuclear uptake of pDNA. Macrophages, in particular, are designed to destroy foreign materials. This adds further complexity to design a delivery system that can bypass these mechanisms. To overcome this, several physiochemical properties must be considered. The shape of NPs has a profound effect on their internalization into macrophages. Specifically, spherically shaped NPs are easily internalized into cells at all points of attachment (50). Second, the surface charge of cationic NPs aids both in the electrostatic attraction of the NPs to the membrane of cells, and to biomolecules such as pDNA for gene transfection. Successfully synthesized NPs with fully cation charged surface were able to load IL-4pDNA, leading to improve the transfection of macrophages. This improves the transfection efficiency and leads to the safe protection of the exogenous genetic molecules (51). Finally, NPs must have a high zeta potential. A low zeta potential indicates NP aggregation, which could prevent efficient transfection. Our data suggests the NPs were spherically shaped and positively charged with a high zeta potential, indicating they have the correct physiochemical properties to transfect primary cells. NPs made by cationic low molecular weight branched PEI lipid provided high transfection and low toxicity, therefore it is a potential therapeutic platform and suitable for biomedical science.

IL-4, an anti-inflammatory cytokine, can trigger M2 macrophage polarization to promote tissue remodeling, wound healing, and immune rebalancing. Thus, treating macrophages with IL-4 to regulate immune signaling pathways may provide significant therapeutic potential for various diseases and disorders. We loaded our NPs with IL-4 pDNA to repolarize M1 macrophages to M2. Human primary macrophages treated with IL-4pDNA-NPs achieved greater than 60% transfection with no cytotoxic effects. MDM cells internalized IL-4pDNA-NPs within 30 minutes, which were then captured in lysosomes. Within 2 hours, lL-4pDNA-NPs escaped the lysosome and entered the nucleus. IL-4 protein expression was observed after 2 days, and continued to express for 9 days, with maximal expression seen at 5 days. The reduced toxicity of our NPs in conjunction with their ability to deliver pDNA to the nucleus of macrophages offers great potential for their use in primary cell research and clinical applications.

Polarization of inflammatory M1 macrophages toward immunosuppressive M2 macrophages is critical for treating many diseases (23, 52, 53). The pro-inflammatory cytokines IL-6, and TNF-α are upregulated in response to inflammatory stimuli including LPS. In contrast, anti-inflammatory cytokine TGF-β is upregulated in response to IL-4. As our data suggests, MDM and BMM cells treated with LPS and IL-4pDNA-NPs show an increase in TGF-β and a decrease in IL-6, and TNF-α, indicating IL-4pDNA-NPs can effectively polarize M1 macrophages to the M2 phenotype. It is likely that the introduction of IL-4 created a positive feedback loop that causes the release of anti-inflammatory cytokines that shut off pro-inflammatory cytokines. In addition, the examination of the M1 marker iNOS and the M2 marker arginase 1 in BMM cells treated with LPS and IL-4pDNA-NPs showed an increase in arginase 1 and a decrease in iNOS. These findings support that IL-4pDNA NPs can affect M1/M2 markers in a manner consistent with M2 polarization.

Studies of NPs delivery *in vivo* is important for understanding the overall effects of pDNA-NPs in a living system. Mice injected with IL-4pDNA-NPs showed no health or behavioral problems, indicating low toxicity. Treatment with IL-4pDNA-NPs caused an increase in IL-4 expression, suggesting IL-4pDNA-NPs were delivered inside the cells and to the nucleus for the production and expression of the IL-4 protein. LPS induced inflammation in mice, followed by IL-4pDNA-NPs treatment resulted in an increase in arginase 1 in mouse tissue macrophages. Arginase 1, a hallmark of M2 macrophages (54), is an enzyme downstream of IL-4 receptor signaling that is involved in the metabolism of arginine to urea and ornithine. It is subsequently used for proline and collagen synthesis to promote wound healing. It is likely the introduction of IL-4 into mouse splenocytes directly caused an overproduction of arginase 1, suggesting IL-4pDNA-NPs can cause M2 polarization. Future studies should investigate the long-term effects of IL-4pDNA-NP treatment including cytotoxicity and the mechanisms underlying wound healing and repair.

We synthesized a new cationic lipid using low molecule PEI to make a core-shell NP for gene delivery. These NPs were capable of optimizing the charge and size to enhance pDNA encapsulation, intracellular transportation, and transfection in macrophages. IL-4pDNA-NPs are able to pass through the cellular and nuclear membranes, and can escape lysosomes for integration into the nucleus. Therefore, this delivery system is capable for macrophages gene-editing. IL-4pDNA-NPs successfully transfected macrophages for the production and secretion of functional IL-4. This led to the repolarization of LPS-induced M1 cells to an M2 phenotype *in vitro*. Importantly, IL-4pDNA-NPs were able to successfully transfect cells *in vivo*, causing increased production of functional IL-4 in splenocytes and the polarization of M1 to M2 macrophages. The ability to manipulate the phenotype of macrophages using IL-4pDNA-NPs provides a novel therapeutic strategy to aid in wound healing and immune rebalancing.

## AUTHOR'S NOTE

Some of the authors have patent applications related to the present study: “High Efficient Delivery of Plasmid DNA into Human and Vertebrate Primary Cells *In Vitro* and *In Vivo* by Nanocomplexes” filed by Texas Tech University, The EFS ID: 39781879 with application #:16956627, International Application No. PCT/US2018/063546 and Application number WO2019/133190.

## Data Availability Statement

The original contributions presented in the study are included in the article/**Supplementary Material**. Further inquiries can be directed to the corresponding author.

## Ethics Statement

All experiments in Balb/C mice were carried out in strict accordance with the recommendations in the Guide for the Care and Use of Laboratory Animals of the National Research Council. The animal study was reviewed and approved by Texas Tech University Health Sciences Center (TTUHSC) Institutional Animal Care and Use Committee.

## Author Contributions

SL and HD designed research. SL performed almost all of experiments and collected the results. JF carry on pDNA encapsulation assay and helped on the data analysis. JD, AB, DW, BC, AT, SM, and JE helped on analysis of flow cytometry and imaging results. JF, TT, and PN-L helped on construction of IL-4/GFP plasmids. HD conceived of the study, developed the theory, contributed to the interpretation of the results, and were in charge of overall direction and planning. Manuscript writing by SL, HD, and JF. All authors contributed to comments and editing. All authors contributed to the article and approved the submitted version.

## Funding

This study was supported by NIH 1R01GM114851-01A1, NIH DK117383, the Seed Grant of Texas Tech University Health Sciences Center El Paso, SABR from Graduate School of Biomedical Sciences of Texas Tech University Health Sciences Center El Paso, and Mini Grant from Paul L. Foster School of Medicine of Texas Tech University Health Sciences Center El Paso.

## Conflict of Interest

The authors declare that the research was conducted in the absence of any commercial or financial relationships that could be construed as a potential conflict of interest.

## Publisher’s Note

All claims expressed in this article are solely those of the authors and do not necessarily represent those of their affiliated organizations, or those of the publisher, the editors and the reviewers. Any product that may be evaluated in this article, or claim that may be made by its manufacturer, is not guaranteed or endorsed by the publisher.

## Supplementary Material

Supplementary Figure 1 IL-4pDNA loading capacity. Encapsulation capacity of IL-4pDNA/GFP-NPs was determined by agarose gel electrophoresis at N/P ratios of 4, 3, 2.6, 2.3, 2, 1.8, and 1.6 (Lane 2-8). Unloaded IL-4pDNA was served as the control (Lane 1). The results indicated that IL-4pDNA-NPs at N/P ratios of 4, 3, 2.6 and 2.3 were fully encapsulated after 30 minutes of incubation. By increasing IL-4pDNA concentration to N/P ratios of 2, 1.8, and 1.6, a positive band was detected on the gel, indicating unencapsulated IL-4pDNA caused by the over-loading.

Supplementary Figure 2 MDM were transfected with IL-4pDNA/GFP-NPs at NPs/media ratios of 1/1, ¼, 1/10, 1/20 and 1/50 for 5 days. The transfected GFP+ MDM were analyzed by flow cytometry.

Supplementary Figure 3 Lysosomes responding to intracellular IL-4pDNA-NPs Lysosomes were labelled with LysoTracker (red). (A) Quantitative analyses of MDM lysosomes showed a gradually increases from 30 min up to 4 hours following IL-4pDNA-NPs treatment. The intensity of lysosomes from IL-4pDNA-NPs treated mouse BMM were examined over 3 days (B). (C) Lysosome distribution in IL-4pDNA-NPs treated mouse BMM exhibited a slower increases up to 1 day, and then a reduction was seen at day 2 and 3.

Supplementary Figure 4 Continuation of IL-4pDNA-NPs transfection in MDM (A) Microscopy imaging of GFP+ MDM illustrated the transfection of IL-4pDNA/GFP-NPs over 25 days. (B) Flow cytometry assay showed the expression of iNOS in MDM response to LPS induced inflammation. (C) IL-4pDNA/GFP-NPs transfection inhibited iNOS expression in LPS induced inflammatory MDM. Error bars represent the standard error of the mean (s.e.m.). * p < 0.01 by comparison to LPS group.

Supplementary Figure 5 IL4/GFP expression in IL-4pDNA-rNPs treated mice.
