# Supplementary figures and images for: Engineering Human Circulating Monocytes/Macrophages by Systemic Deliverable Gene Editing
Source: Front Immunol. 2022 May 18;13:754557. doi: 10.3389/fimmu.2022.754557 (PMC9159279; doi:10.3389/fimmu.2022.754557)

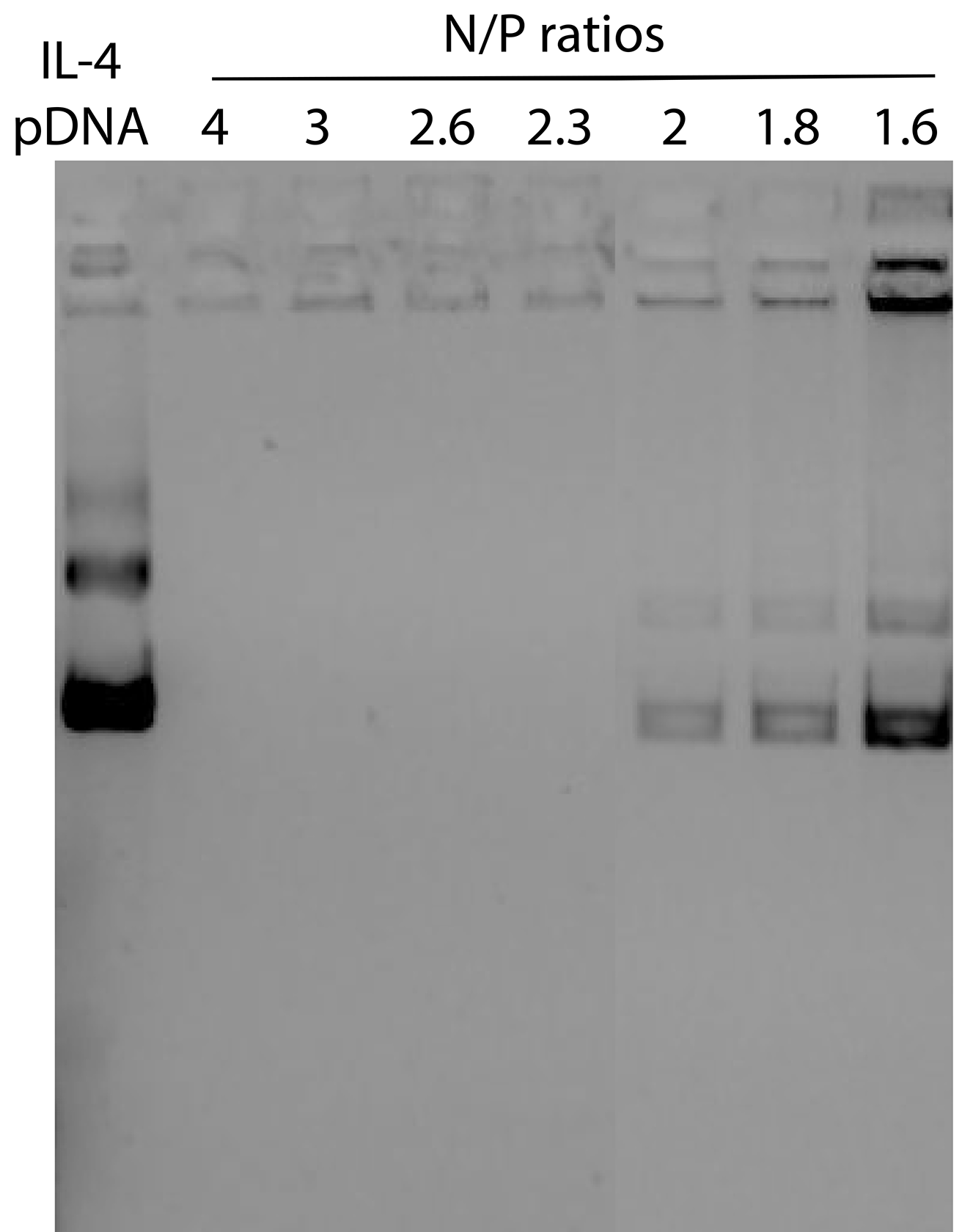

Supplement: Supplementary Figure 1 — IL-4pDNA loading capacity. Encapsulation capacity of IL-4pDNA/GFP-NPs was determined by agarose gel electrophoresis at N/P ratios of 4, 3, 2.6, 2.3, 2, 1.8, and 1.6 (Lane 2-8). Unloaded IL-4pDNA was served as the control (Lane 1). The results indicated that IL-4pDNA-NPs at N/P ratios of 4, 3, 2.6 and 2.3 were fully encapsulated after 30 minutes of incubation. By increasing IL-4pDNA concentration to N/P ratios of 2, 1.8, and 1.6, a positive band was detected on the gel, indicating unencapsulated IL-4pDNA caused by the over-loading. [file Image_1.tif]

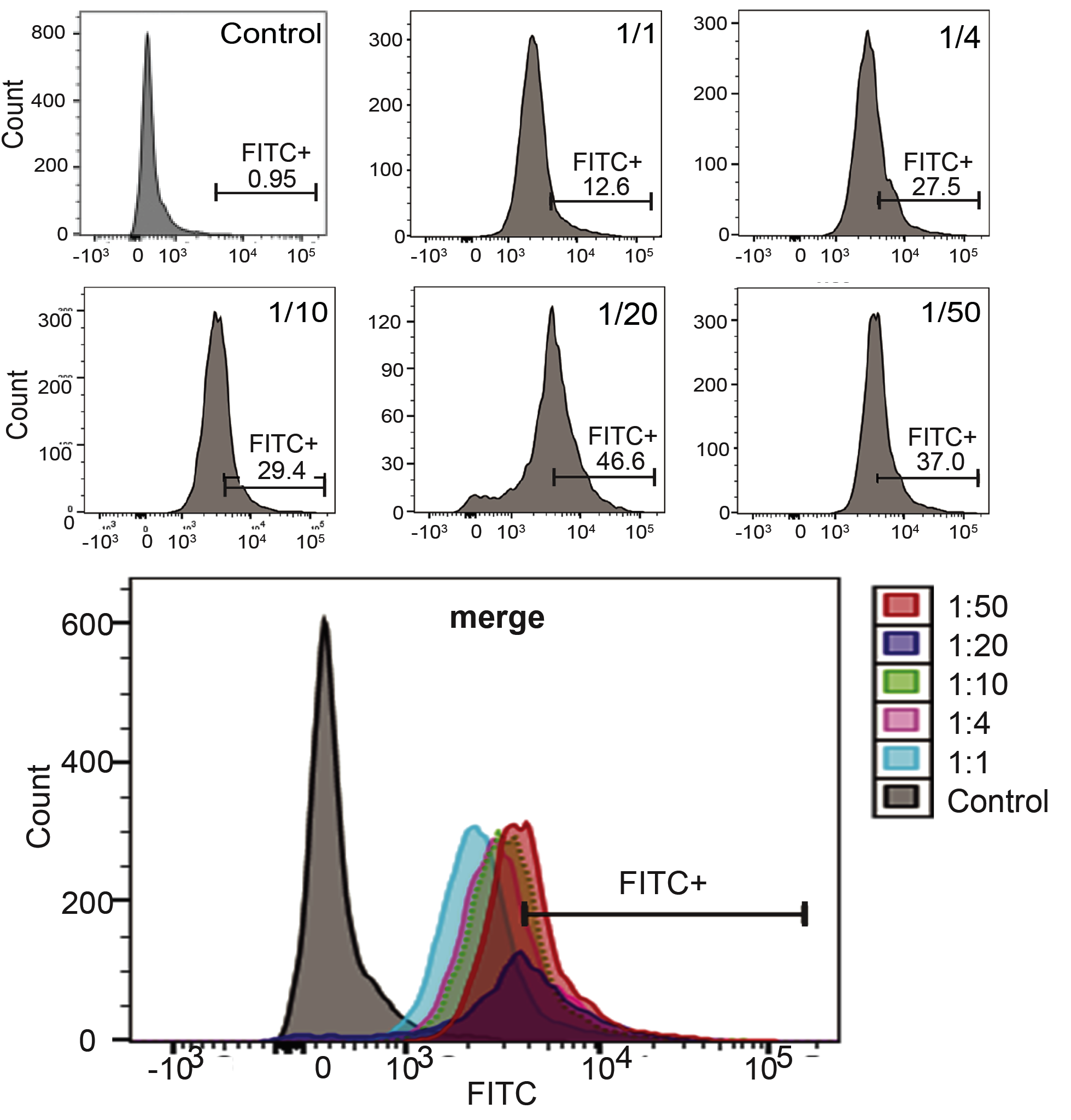

Supplement: Supplementary Figure 2 — MDM were transfected with IL-4pDNA/GFP-NPs at NPs/media ratios of 1/1, ¼, 1/10, 1/20 and 1/50 for 5 days. The transfected GFP+ MDM were analyzed by flow cytometry. [file Image_2.tif]

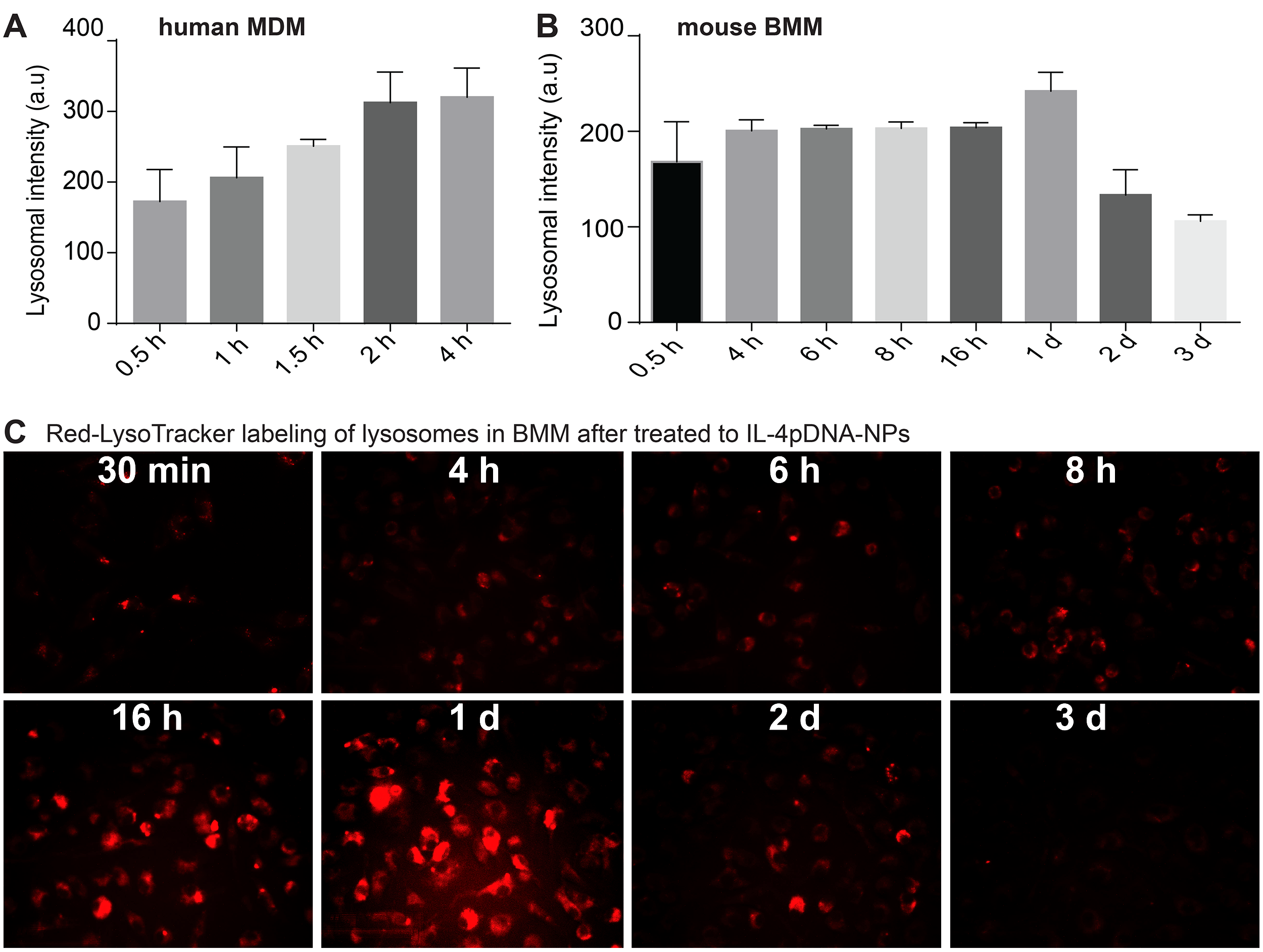

Supplement: Supplementary Figure 3 — Lysosomes responding to intracellular IL-4pDNA-NPs Lysosomes were labelled with LysoTracker (red). (A) Quantitative analyses of MDM lysosomes showed a gradually increases from 30 min up to 4 hours following IL-4pDNA-NPs treatment. The intensity of lysosomes from IL-4pDNA-NPs treated mouse BMM were examined over 3 days (B). (C) Lysosome distribution in IL-4pDNA-NPs treated mouse BMM exhibited a slower increases up to 1 day, and then a reduction was seen at day 2 and 3. [file Image_3.tif]

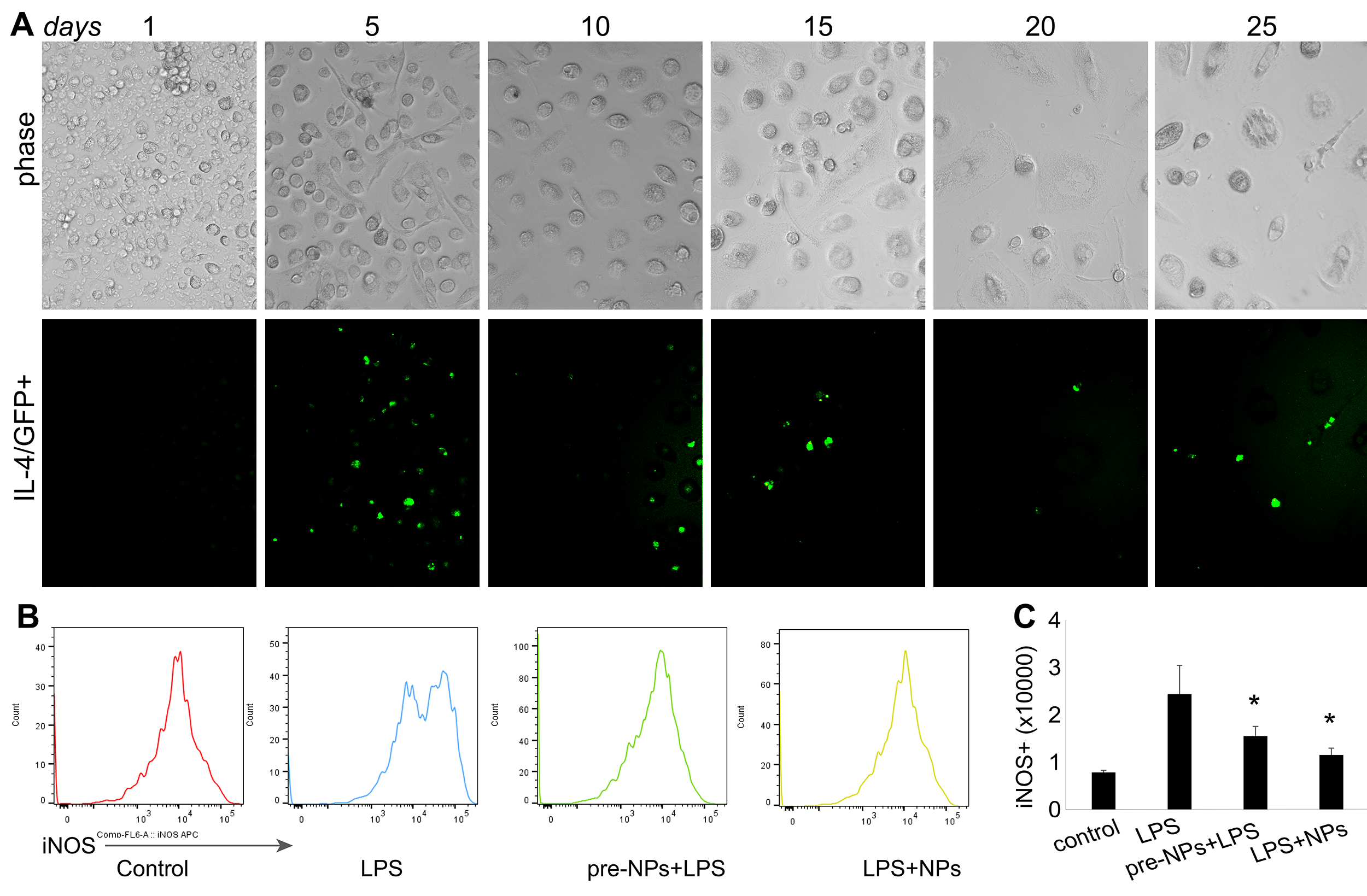

Supplement: Supplementary Figure 4 — Continuation of IL-4pDNA-NPs transfection in MDM (A) Microscopy imaging of GFP+ MDM illustrated the transfection of IL-4pDNA/GFP-NPs over 25 days. (B) Flow cytometry assay showed the expression of iNOS in MDM response to LPS induced inflammation. (C) IL-4pDNA/GFP-NPs transfection inhibited iNOS expression in LPS induced inflammatory MDM. Error bars represent the standard error of the mean (s.e.m.). * p < 0.01 by comparison to LPS group. [file Image_4.tif]

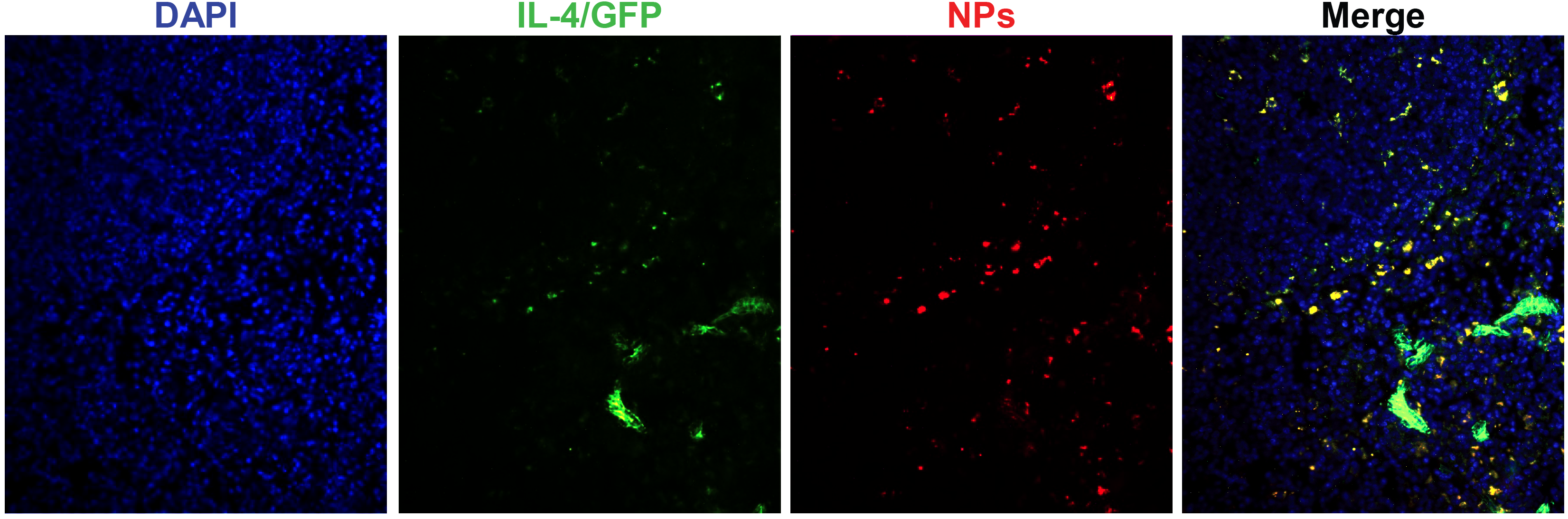

Supplement: Supplementary Figure 5 — IL4/GFP expression in IL-4pDNA-rNPs treated mice. [file Image_5.tif]
